# Incidence and Burden of the Myelodysplastic Syndromes

**DOI:** 10.1007/s11899-015-0269-y

**Published:** 2015-07-02

**Authors:** Christopher R. Cogle

**Affiliations:** Division of Hematology and Oncology, Department of Medicine, College of Medicine, University of Florida, 1600 SW Archer Road, Box 100278, Gainesville, FL 32610-0278 USA

**Keywords:** Myelodysplastic syndromes, Epidemiology, Incidence, Prevalence

## Abstract

Since 2001, cases of myelodysplastic syndromes (MDSs) have been tracked by cancer registries. Examining registry data in the USA, the reported age-adjusted incidence of MDS per 100,000 was 3.3 per year for 2001–2003 and 4.9 per year for 2007–2011, with increases likely a result of growing awareness of reporting requirements. However, active case-finding methods repeatedly demonstrate that population-based registries have underestimated the incidence of MDS due to underreporting and underdiagnosis. Using keyword search strategies of electronic pathology reports or other novel case capture methods, the true incidence of MDS has been estimated between 5.3 and 13.1 per 100,000. Using Medicare billing claims data, the incidence of MDS per 100,000 in patients aged ≥65 years has been estimated between 75 and 162. MDS prevalence is estimated to be 60,000 and –170,000 in the USA and projected to grow. Epidemiologic data can help estimate the burden of MDS and expose unmet clinical needs. For example, patients with MDS receiving transfusions had significantly higher reported health care costs versus those that did not (3-year mean of $88,824 vs $29,519). Epidemiologic data also revealed that most MDS patients receiving transfusions do not receive active therapies, despite strong evidence that hypomethylating agents and lenalidomide significantly reduce transfusion burden. Other unmet needs identified by epidemiologic studies include high need for treatment options after failing first-line therapy and shared decision making by older MDS patients.

## Introduction

The myelodysplastic syndromes (MDSs) are a diverse group of clonal hematopoietic malignancies characterized by ineffective hematopoiesis, progressive bone marrow (BM) failure, cytogenetic and molecular abnormalities, and variable risk of progression to acute myeloid leukemia (AML) [1–3]. Recent reports showed that 5–10 % of older, apparently healthy individuals had acquired ≥1 myeloid gene mutation, whereas younger individuals were much less likely to have acquired clonal hematopoiesis with somatic mutations [4, 5]. These results support the notion that the origin of MDS is tied to aging and provide biological rationale for why MDS most often presents in the seventh and eight decades of life [6, 7]. Clinically, patients with MDS often present with symptoms related to peripheral cytopenias (e.g., fatigue, pallor, infections, bruising, bleeding), though they may also be asymptomatic with abnormal blood counts found on routine evaluation [8]. In elderly patients, cytopenias due to MDS—particularly anemia—may be attributed to a more indolent etiology [9].

In the ninth edition of the International Classification of Diseases (ICD-9), MDS was coded as a disease of blood and blood-forming organs. In the tenth edition (ICD-10), MDS was reclassified as a neoplasm and thus also included in the third edition of ICD for Oncology (ICD-O-3), which provide clarity in MDS epidemiology (Table 1). The accuracy of determining MDS incidence and prevalence is crucial to allow for better understanding of the burden of MDS and appropriate allocation of heath care resources.

**Table 1:** Coding of MDS using ICD-O-3 and ICD-9-CM

	RAEB-2	RAEB-1	RARS	RA	RCMD	RCMD-RS	t-MDS NOS	MDS 5q	MDS NOS
ICD-O-3	9983^a^		9982	9980	9985		9987^b^	9986	9989
ICD-9-CM									
Before Oct 2006	285.0			284.9	283.7				
Oct 2006–Oct 2009	238.73		238.72					238.74^c^	238.75
Oct 2009–Oct 2015^d^	238.73	238.72						238.74^c^	238.75

*5q* 5q deletion syndrome, *MDS* myelodysplastic syndromes, *NOS* not otherwise specified, *RA* refractory anemia, *RAEB* refractory anemia with excess blasts, *RAEB-1* RAEB with 5–9 % blasts, *RAEB-2* RAEB with 10–19 % blasts, *RARS* refractory anemia with ringed sideroblasts, *RCMD* refractory anemia with multilineage dysplasia, *RCMD-RS* RCMD with ringed sideroblasts, *t-MDS* therapy-related MDS

^a^Prior to 2010, RAEB-in transformation had a unique code (9984) but is now grouped with RAEB-1

^b^Prior to 2010, t-MDS had a unique code (9987) but is now grouped with other therapy-related myeloid neoplasms

^c^Excludes patients with high-grade MDS with 5q deletion, which are coded as 238.72

^d^As of October 1, 2015, ICD-10-CM with replace ICD-9-CM

## Estimated Incidence of MDS

### Population-Based Registries

Implementation of ICD-O-3 occurred worldwide in 2001, from which time captured cases of MDS could be tracked via cancer registries. The initial report from National Cancer Institute Surveillance, Epidemiology, and End Results (SEER) estimated the age-adjusted incidence of MDS per 100,000 to be 3.28, 3.37, and 3.56 in 2001, 2002, and 2003, respectively (Table 2) [6]. Estimated rates per 100,000 per year (2001–2003) in older patients increased from 10 in those aged 65–69 years to 36.4 in those aged ≥85 years. Shortly after, a more comprehensive report from the North American Association of Central Cancer Registries (NAACCR) and SEER estimated the age-adjusted incidence to be 3.27 per 100,000 per year (2001–2003) [10]. Rates per 100,000 per year in older patients increased from 7.14 in those aged 60–69 years to 35.49 in those aged ≥80 years. Estimated age-adjusted incidence per 100,000 significantly increased by year, from 3.1 in 2001 to 3.8 in 2004. Increasing incidence estimates from 2001 to 2004 are likely a result of improved awareness of the requirement of reporting resulting in rising capture rates during these early years of ICD-O-3. However, in the NAACCR/SEER study, only 4 % of MDS cases were reported to registries from physicians’ offices [10]. Since MDS is often diagnosed and managed outside of a hospital setting, this surprisingly low percentage of cases reported from outpatient clinics begged the question whether MDS cases were being markedly underreported to population-based cancer registries. Furthermore, since estimated MDS incidence rates reported from various European countries (including the UK [16], Germany [17, 18], Sweden [19], France [20]), and Australia [21] were similar, underreporting was suspected worldwide.

**Table 2:** Summary of estimated incidence rates of MDS in the USA

Data sources	Age-adjusted incidence per 100,000 per year	Incidence in elderly populations per 100,000 per year
SEER Registry 2001–2003 ICD-O-3 for MDS (9980–9989) [6]	3.28 in 2001 3.37 in 2002 3.56 in 2003	65–69 years, 10 70–74 years, 16.6 75–79 years, 25.7 80–84 years, 36.2 ≥85 years, 36.4
NAACCR/SEER Registries 2001–2004 ICD-O-3 for MDS (9980–9989) [10]	3.27 per year (2001–2003) 3.10 in 2001 3.29 in 2002 3.45 in 2003 3.8 in 2004 (SEER data only)	60–69 years, 7.14 70–79 years, 20.05 ≥80 years, 35.49
SEER Registry 2007–2011 ICD-O-3 for MDS (9980–9989) [11]	4.9 per year (2007–2011) 2007, 5.07 2008, 4.85 2009, 4.94 2010, 5.13 2011, 4.70	65–69 years, 13.47 70–74 years, 23.89 75–79 years, 37.93 80–84 years, 53.16 ≥85 years, 63.62
2003 Medicare Standard Analytic Files ICD-9-CM for MDS (238.7x) [12]	NA	≥65 years, 162 65–69 years, 114 70–74 years, 146 75–79 years, 176 ≥80 years, 204
SEER-Medicare database 2000–2008 ICD-9-CM for MDS (285.0, 238.7, 284.9 until Oct 2006; 238.72–238.75 for Oct 2006–2008) *with* ICD-O-3 for BC (CPT 85004, 85007–85009, 850013, 850014, 850018, 850021–85027, 85031, 85032, 85041, 85044–85049, 85060, 85590, 85595, G0306, G0307) and ICD-O-3 for BM biopsy/aspiration (CPT 38220, 38221, 85095, 85097, 85102, G0364 or ICD-8-CM procedure 413.1 and 413.8) [13]	NA	≥65 years, 75 (using 2 + BCBM algorithm) 2+ BCBM algorithm required two ICD-9-CM claims within 12 months (or death/hospice within 3 months of first claim) and BC and BM biopsy in the year prior to first claim.
Florida Cancer Data System registry 2006 ICD-O-3 for MDS (9980–9989) *with* Electronic pathology reports with BM biopsies Keyword search strategy [14•]	5.3 per year	NR
Seattle-Puget Sound SEER registry region 2005–2006 ICD-O-3 for MDS: 9980–9989 *with* Group Health Cooperative health care data ICD-9-CM for MDS (238.7x) and medical chart review [15]	SEER alone, 6.9 SEER + definite/probable MDS by chart review, 7.0 SEER + definite/probable/possible MDS by chart review, 10.2	NR

*BC* blood count; *BM* bone marrow; *ICD-9-CM* International Classification of Diseases Ninth Edition-Clinical Modification; *ICD-O-3* International Classification of Diseases for Oncology Third Edition; *MDS* myelodysplastic syndromes; *NA* not applicable; *NAACCR* North American Association of Central Cancer Registries; *NR* not reported; *SEER* Surveillance, Epidemiology, and End Results

More recent data from SEER (2007–2011) estimated the age-adjusted incidence to be 4.9 per 100,000 per year [11]. Estimated rates per 100,000 per year in elderly patients increased from 13.5 in those aged 65–69 years to 63.6 in those aged ≥85 years. Interestingly, the previous trend in estimated rates from registry data increasing each year was no longer observed, as the estimated age-adjusted incidence rate was highest at 5.1 per 100,000 in 2007 and 2010 and lowest at 4.7 per 100,000 in 2011. Despite this apparent plateau, estimates likely still underrepresent true incidence, as many cases of MDS are not reported to cancer registries due to underdiagnosis, lack of recognition of MDS as a malignancy, limited reporting by outpatient clinics, and changing guidelines for coding of MDS cases [13, 14•, 22, 23]. It has been suggested that passive case-finding methods such as registries may capture fewer patients with MDS than active case-finding methods such as those using billing claims data, pathology, cytogenetic, or other laboratory testing records [24].

### Billing Claims-Based MDS Case Ascertainment

Use of Medicare billing claims data limits analyses to patients aged ≥65 years; however, this cohort includes the great majority of MDS cases. Medicare claims represent services provided across specialties and care settings, which may address issues with low registration of patients in outpatient settings. By retrospective review of 2003 Medicare Standard Analytic Files using ICD-9-Clinical Modification (CM) code 238.7x to identify patients with newly diagnosed MDS, Goldberg and colleagues estimated the incidence to be 162 per 100,000 in patients aged ≥65 years (114 in those aged 65–69 years to 204 in those aged ≥80 years) [12]. This estimate is approximately fourfold to fivefold higher compared to those from SEER and NAACCR registry reports from similar time frames [6, 10]. Use of the nonspecific ICD-9-CM code 238.7x to identify MDS cases could account for some this increase. ICD-9-CM codes for billing are selected by treating physicians and serve as their impression of the diagnosis rather than a confirmed pathological diagnosis, while registries rely on pathologically derived ICD-O-3 codes. The ICD-9-CM has undergone several changes in coding for MDS (Table 1), which impacts estimates of MDS incidence. Therefore, publications using ICD-9-CM should be clear about dates of examination and specific ICD-9-CM codes used.

To address the specificity issue and improve case identification, we developed four claims-based algorithms and assessed algorithm validity using MDS cases registered by SEER [13]. The 1+ algorithm required a single claim with ICD-9-CM diagnosis of MDS, which aligned with the analysis from Goldberg and colleagues [12]. The 2+ algorithm also required a second claim 1–12 months after the first claim or death/hospice entry within 3 months of the first claim. The 2+ BC algorithm also required a blood count (BC) in the year before the first claim; during this time period, guidelines for MDS diagnosis (World Health Organization [WHO] and French-American-British [FAB]) required a BC. Lastly, the 2+ BCBM algorithm also required both a BC and bone marrow (BM) biopsy in the year before the first claim; during this time period, guidelines for MDS diagnosis (WHO/FAB) recommended but did not require a BM biopsy. Algorithms were applied to the 2000–2008 SEER-Medicare database. The 2+ BCBM algorithm was the most specific claims-based strategy and estimated an MDS incidence of 75 per 100,000 in patients ≥65 years in 2005—compared to 20 per 100,000 reported by SEER.

While a large sample size is a clear strength of claims-based data capture, this approach limits the ability to examine details of patient demographics and disease characteristics due to lack of clinical information in administrative datasets. Additionally, use of ICD-9-CM codes is limited to insured populations and excludes patients enrolled in hospice or managed care organizations that do not generate claims.

### Population-Based Case Ascertainment

Using 2006 data from the Florida Cancer Data System (FCDS) as a model, we devised a keyword search strategy to identify cases of MDS among electronic pathology (e-path) reports received by cancer registries from pathology laboratories [14•]. Since pathology laboratories are legislatively mandated to transmit cancer pathology reports to paper-based state cancer registries, our keyword strategy was expected to depend less on the physical location of the patient’s diagnosis and identify more potential cases. The search was restricted to electronic reports with BM biopsy by querying on the term “marrow.” It was found that 38 % of MDS cases identified from e-path reports were not linked to an existing paper case in the FCDS registry. Since paper-based registry data from NAACCR/SEER estimated the age-adjusted incidence in Florida to be 3.94 per 100,000 per year (2001–2003) [10], including the uncaptured cases found by e-path reports extrapolated the estimated MDS incidence to 6.4 per 100,000 per year in Florida and 5.3 per 100,000 per year in the entire USA.

Within a nonprofit health care system in western Washington State (2005–2006), MDS cases were identified via SEER registry or relevant diagnostic code, followed by medical chart review [15]. This region had reported the highest rates of MDS among the SEER registry regions since mandatory reporting began in 2001. This could reflect a true regional difference or a false difference due to incomplete case ascertainment or underdiagnosis varying by region. When combining cases from SEER with those identified by medical chart review as “definite/probable” cases of MDS (BM biopsy or cytogenetics indicating MDS or physician’s notes regarding MDS diagnosis), the estimated age-adjusted incidence rate was 7.0 per 100,000 per year. This was very similar to the 6.9 per 100,000 per year reported using SEER alone, suggesting that reporting to SEER in this region was nearly complete during this time frame. However, incidence is likely still underestimated due to omission of cases that did not receive definitive diagnoses of MDS, as the addition of those identified by medical chart review as “possible” MDS cases (documentation that MDS was suspected or never definitively ruled out) raised the estimated age-adjusted incidence rate to 10.2 per 100,000 per year.

A retrospective study of diagnostic and procedure data from >300 hospitals in the Australian state of Victoria (1998–2008) also showed a higher estimated incidence of MDS (9.6 per 100,000) compared to Australia cancer registry data (4.8 per 100.000) in 2007 [25]. Hospitalization data (from Victorian Admitted Episode Dataset [VAED]) was subsequently linked with data from the statewide Victorian Cancer Registry to examine patients with MDS from 2003 to 2010 [26•]. Cases of MDS were identified in VAED by diagnostic code D46 from the Australia Modification of ICD-10. The majority (86 %) of registry-reported cases were linked to VAED data. For those not linked, most cases had been reported to registry by pathology laboratories and patients may not have been hospitalized. However, approximately half of VAED cases were not reported to registry. Using registry data only, the estimated age-adjusted incidence was 6.3 per 100,000 per year and 44 per 100,000 per year in those aged ≥65 years. When pooling registry and VAED data, the estimated age-adjusted incidence increased to 10.1 per 100,000 per year and 68 per 100,000 per year in those aged ≥65 years. Next, a capture-recapture technique [27] was used to estimate the extent of incomplete case ascertainment from the overlapping registry and VAED datasets [26•]. With multinomial logistic regression applied, the estimated age-adjusted incidence was further increased to 13.1 per 100,000 per year and 103 per 100,000 per year in those aged ≥65 years.

With several methods tested and validated for capturing cases of MDS, investigators have options. The choice of methodology depends on the completeness of registry data and available search fields. Going forward, new search engine and mapping technologies could be applied to e-path reports sent from diagnostic pathology laboratories to cancer registries.

## Estimated Prevalence of MDS

Accurate estimates of the number of people living with MDS are difficult to make, and prevalence is scarcely reported. Using Düsseldorf MDS Registry data from 1996 to 2005, the overall prevalence of MDS was estimated to be 7 per 100,000 [18]. Some investigators have extrapolated these German data and estimated ≈60,000 people living with MDS in the USA [28, 29]. Considering that registry incidence estimates are low, it is probable that this prevalence rate is also an underestimate. Data from the third National Health and Nutrition Examination Survey (1988–1994) showed that 10.6 % of the noninstitutionalized US population aged ≥65 years had anemia [30]. Approximately, a third of these patients had unexplained anemia, 17 % of which had hematologic features consistent with MDS (macrocytosis, neutropenia, or thrombocytopenia). Sekeres and colleagues extrapolated this figure and estimated 170,000 people living with MDS in the USA [28]. However, as there were no confirmatory BM evaluations, this may be an overestimation.

With that in mind, another group retrospectively examined BM from 322 patients at their Canadian center with unexplained cytopenias [31]. Of these, 23 % had a confirmed diagnosis of MDS and 10 % had a suspected diagnosis (element of dyserythropoiesis, dysmegakaryopoiesis, or dysgranulopoiesis detected but complete criteria for diagnosis not fulfilled or secondary causes not yet excluded). Extrapolating their data, they estimated >90,000 people ≥65 years living with MDS in the USA. However, measuring the frequency of MDS only in patients in whom a BM biopsy was indicated may also bias the sample. The true prevalence of MDS in the USA likely lies somewhere between the estimates of 60,000 and 170,000, though prevalence will continue to rise as more active therapies are available and patients are living longer with MDS.

## Using Epidemiologic Tools to Estimate Burden of MDS and Expose Unmet Needs

### US Health Care Costs

To estimate health care resource utilization for patients with MDS, analysts need accurate identification of MDS cases and costs of medications, transfusions, procedures, and hospitalizations associated with the disease. Using National Comprehensive Cancer Network guidelines for treating patients with lower-risk MDS, the cost of drugs alone was projected to average $63,577 per patient annually [32]. When examining actual costs (excluding outpatient prescription drugs), a retrospective review of Medicare Standard Analytic Files showed that Medicare payments for patients with MDS were significantly higher than non-MDS Medicare beneficiaries (means of $25,834 vs $6,810 in 2003, $19,180 vs $7,438 in 2004, and $18,758 vs $7,910 in 2005) [12].

In a multiple regression model, the presence of certain baseline characteristics, clinical complications, and need for transfusions were predictive, to varying degrees, of increases in cost [33•]. With adjustment for baseline characteristics and clinical complications, patient costs increased 77 % with dyspnea, 71 % with sepsis, 51 % with arrhythmia, 49 % with bacteremia, 48 % with transfusions, 43 % with congestive heart failure, 32 % with history of heart problems, and 30 % with pneumonia. Of note, for patients who required transfusions, this cost increase did not include the additional costs related to transfusion administration. Patients receiving transfusions also had greater use of hospital inpatient and outpatient services and significantly higher Medicare costs (3-year mean of $88,824 vs $29,519 for nontransfused patients) [33•]. Other studies have also examined the economic impact of transfusion dependence (TD) and found markedly greater use of inpatient and outpatient services and consequently significantly higher costs [34, 35]. A literature search of Medline and Embase found that reported annual medical costs per patient with MDS ranged from $9,840 to $19,811 for those who were transfusion independent (TI) vs $29,608-$51,066 for those who were TD [35].

### Iron Overload

In patients with MDS, severe anemia resulting in red blood cell (RBC)-TD was associated with increased mortality and decreased quality of life [35–38]. Transfusions can lead to iron overload, which associated with morbidities and adversely impacted survival [36, 39]. A retrospective review of Medicare Standard Analytic Files showed that during 3 years of follow-up—even with age adjustment—MDS significantly associated with increased risk of iron overload-related complications (cardiac-related events, diabetes, dyspnea, hepatic diseases, and infections) compared to the general Medicare population [12]. Unsurprisingly, in the 40 % of patients that received transfusions in this assessment, each of these complications was significantly more prevalent than in non-transfused patients with MDS. Patients receiving transfusions also had significantly shorter overall survival (OS), increased risk of transformation to AML, and increased risk of death.

A separate retrospective review of a US health insurance claims database (1997–2004) also showed that receipt of RBC transfusions significantly increased the risk of iron overload-related complications including cardiomyopathy/heart failure, conduction/rhythm disorders, diabetes, and liver disease [40]. Hospitalization data from the Australian state of Victoria also showed that RBC-TD patients had significantly higher rates of congestive heart failure, bacterial and fungal infections, transformation to AML, and sepsis as cause of death [25].

More recently, a retrospective claims review was performed using the Optum Research Database (2007–2009), which includes medical and pharmacy claims and eligibility information from a national US health plan [41•]. Of 4351 patients with MDS identified, 1105 received ≥1 transfusion, and transfusions were associated with higher risk of infection, bleeding events, hospitalizations, and emergency room visits. Patients who had active therapies but no transfusions had lower risks of these events than patients who had transfusions with or without active therapies. In patients that had transfusions, the risks of infection or bleeding events were similar whether or not they had active therapy. Surprisingly, most patients (*n* = 886) receiving transfusions were not receiving active therapies [41•], although active therapies such as azacitidine and lenalidomide have been shown to significantly reduce RBC transfusion burden [42, 43]. A study of lenalidomide asserted that this reduction extends to cost burden—as the cost of lenalidomide treatment was more than offset by the savings related to reductions in TD and the associated complications [44]. The low proportion of patients with MDS receiving active therapies despite requiring transfusions exposes a clinical practice mismatch worthy of further investigation. Are patients with MDS consistently being offered active treatments? Are patients with MDS often refusing active treatments? Are physicians uncomfortable treating patients with MDS using agents that may transiently worsen cytopenias?

### Active Treatment of Older Patients

Some older patients with MDS may not receive active treatment due to age-related comorbidities and functional impartment or the perception that therapies will not extend their survival. A prospective study of 43 patients aged ≥60 years with high-risk MDS or AML showed that treatment with intensive chemotherapy (IC) was significantly associated with younger age but not related to performance status, comorbidities, or quality of life [45]. Lack of treatment with IC in clinical practice is often largely subjective, a result of patient or physician opinion, or patient refusal.

However, newer lower-intensity therapies have demonstrated efficacy and tolerability in the older population. Azacitidine has been shown to significantly prolong OS vs conventional care regimens (CCR; IC, low-dose cytarabine, or best supportive care [BSC]) in patients with high-risk MDS [42], including a subset of patients aged ≥75 years [46]. Decitabine has demonstrated significantly improved rates of responses and hematologic improvement in patients with MDS [47] and, in patients aged ≥60 years with higher-risk MDS, significantly prolonged progression-free survival and time to AML transformation [48]. With lenalidomide treatment, the majority of patients with lower-risk, RBC-TD, del(5q) MDS achieve RBC-TI and cytogenetic response [49], at significantly higher rates than with placebo [43]. A retrospective analysis of patients ≥75 vs <75 years old treated with lenalidomide showed similar rates of response and time to progression to AML [50].

A population-based study using the SEER-Medicare database examined the use of hypomethylating agents (HMA) azacitidine and decitabine in older patients (>65) with MDS during the introductory years for these agents (*N* = 4416; diagnosed from 2001 to 2005) [51]. Overall, 11 % of patients had received HMAs by the end of 2007 and frequency of chemotherapy use was lower than HMA use throughout the study period. Additionally, younger patients were more likely to receive HMAs than older patients. A collection of six cross-sectional surveys including 101 US hematologists and oncologists (June 2005–January 2007) showed that 27 % of recently diagnosed MDS patients (*n* = 198, six surveys pooled) and 24–49 % of established MDS patients *(n* = 4514, six surveys separate) received supportive care only [52]. A retrospective analysis of US claims data (January 2007–June 2010) including patients aged ≥65 years with ≥2 claims with ICD-9-CM diagnosis code for MDS (N = 3577) showed that just 13 % had received any active therapy for MDS [53]. Additionally, active therapy use decreased with age—regardless of Charlson comorbidity index score. Together, these preliminary findings show that despite the availability of active treatments for MDS, a large proportion of older patients forgo treatment, possibly due to patient or physician reluctance. This is an unresolved issue that requires investigation.

### Need for Therapies After HMA Failure

A population-based study using the SEER-Medicare database showed that patients who received HMAs had significantly improved 24-month survival versus patients that did not [54]. However, for patients who did not respond to HMAs, or those who relapsed or progressed after response, prognosis was poor [55–58]. Due to lack of treatment options, current guidelines suggest clinical trial or consideration of allogeneic stem cell transplant in patients following HMA failure [59]. Guidelines also assert that HMA therapy should be continued at least four to six cycles before assessing for failure. A limited case series showed that decitabine treatment following azacitidine failure yielded modest response rates (0–28 %) that are typically brief [57, 60–63].

Recently, a US commercial health insurance claims database was used to examine subsequent treatment patterns for patients with MDS who had received HMAs [64, 65]. Patients with ICD-9-CM diagnosis code 238.7x were identified who used an HMA during 2009–2011. Patients were eligible for second-line therapy after they had used the same HMA for >7 months, discontinued treatment for ≥2 months, or switched to another HMA. Of 1366 patients identified, 402 were eligible for second-line therapy. Approximately 70 % and 30 % of patients received azacitidine and decitabine in the first line, respectively, and the mean all-cause and MDS-specific annual health care costs during first-line treatment were $127,162 and $80,673, respectively. Nearly half (48 %) of patients stopped or switched HMA treatment at <5 treatment cycles. The majority (61 %) of patients received BSC in the second line; 30, 18, and 5 % received azacitidine, decitabine, or lenalidomide, respectively. Following first-line treatment failure, for those eligible for second-line therapy, the mean all-cause 6-month health care cost was $76,945. A separate retrospective study of patients with ICD-9-CM code 238.7x from 2009 to 2011 also showed that approximately 70 % of patients treated with HMAs received <6 cycles; 32 % stopped therapy after only 1 cycle [66]. These results highlight a large and costly unmet need in patients with MDS who fail first-line HMA treatment. There is a clear and urgent need for second-line agents to treat patients with MDS.

### Risk Assessment in Patients with MDS

The treatment paradigm for patients with MDS depends upon prognostic risk assessment [7, 59, 67–69]. However, applying prognostic models to epidemiologic data is not possible, as clinical and laboratory variables such as hemoglobin level or genomic mutations are often not captured in population-based registries or billing claims datasets. Recently, the 2001–2007 SEER-Medicare dataset was used to derive a completely claims-based prognostic scoring system termed the SEER-Medicare MDS Risk Score (SMMRS) [70]. Risk predictors in SMMRS were cytopenias, MDS pathologic subtype, age at diagnosis, Charlson comorbidity index score, presence of acute hospitalizations, and RBC- and/or platelet-TD. The MDS clinical database from the Dana-Farber Cancer Institute was used for external validation of the SMMRS and showed that risk stratification by SMMRS was not significantly different than by International Prognostic Index score [67]. While the SMMRS is not intended for clinical use, it has potential utility in research using the SEER-Medicare dataset, for example, to help address potential impacts of confounding due to baseline disease risk.

### Identifying Populations at Risk for Developing MDS

With approximately 30 % of MDS cases arising after chemotherapy or radiotherapy (RT) treatment of an antecedent cancer [71], there is urgent need to identify patients with solid tumors that are at higher risk for therapy-related MDS. Recently, Mukherjee and colleagues combined registry databases and discovered that modern RT techniques did not increase the risk of developing MDS in patients with prior history of prostate cancer [72, 73]. These results added to mounting evidence that modern RT alone may not trigger therapy-related MDS, leaving investigators to focus on other factors such as age, DNA repair activity, and health of BM microenvironment.

## Conclusions

Since 2001, cases of MDS have been legislatively mandated for tracking in cancer registries. Registry data in the USA and elsewhere have provided estimates of MDS incidence; however, billing claims data and other active case-finding methods have repeatedly demonstrated that population-based registries have underestimated the incidence of MDS. This underreporting highlights the urgent need for deeper investment in state cancer registries. Incidence estimates are further hindered by underdiagnosis of MDS in older patients with cytopenias. With rapidly evolving estimates of incidence, estimating prevalence is difficult, particularly with the addition of active therapies that can help patients with MDS live longer. As epidemiologic data for patients with MDS becomes more robust, it has the potential to be used to identify and/or help address unmet needs for patients
